# Distinguish the Value of the Benign Nevus and Melanomas Using Machine Learning: A Meta-Analysis and Systematic Review

**DOI:** 10.1155/2022/1734327

**Published:** 2022-10-14

**Authors:** Suli Li, Yihang Chu, Ying Wang, Yantong Wang, Shipeng Hu, Xiangye Wu, Xinwei Qi

**Affiliations:** ^1^First Affiliated Hospital of Xinjiang Medical University, China; ^2^Central South University of Forestry and Technology, China; ^3^CHD University, China

## Abstract

**Background:**

Melanomas, the most common human malignancy, are primarily diagnosed visually, beginning with an initial clinical screening and followed potentially by dermoscopic analysis, a biopsy, and histopathological examination. We aimed to systematically review the performance and quality of machine learning-based methods in distinguishing melanoma and benign nevus in the relevant literature.

**Method:**

Four databases (Web of Science, PubMed, Embase, and the Cochrane library) were searched to retrieve the relevant studies published until March 26, 2022. The Predictive model Deviation Risk Assessment tool (PROBAST) was used to assess the deviation risk of opposing law.

**Result:**

This systematic review included thirty researches with 114007 subjects and 71 machine learning models. The convolutional neural network was the main machine learning method. The pooled sensitivity was 85% (95% CI 82–87%), the specificity was 86% (82–88%), and the *C*-index was 0.87 (0.84–0.90).

**Conclusion:**

The findings of our study showed that ML algorithms had high sensitivity and specificity for distinguishing between melanoma and benign nevi. This suggests that state-of-the-art ML-based algorithms for distinguishing melanoma from benign nevi may be ready for clinical use. However, a large proportion of the earlier published studies had methodological flaws, such as lack of external validation and lack of clinician comparisons. The results of these studies should be interpreted with caution.

## 1. Introduction

Melanoma has the highest mortality rate of any type of skin cancer worldwide, especially among Caucasians [1]. Early diagnosis of melanoma significantly reduces mortality and complications.

Previously, differentiating melanoma from benign moles required years of diagnostic experience. With the rapid development of artificial intelligence (AI) in image classification in recent years, images of melanoma and benign nevus can be distinguished by using DL/ML [2]. Melanoma has become a serious health issue due to its increased incidence. Early detection and treatment are of great necessity to reduce the mortality rate. Thus, accumulating research investigated accurate algorithmic models for identification to assist in the early detection and diagnosis of melanoma [3].

Traditional machine learning (before deep neural networks) has made significant progress in identifying medical images in recent years [4]. The World Health Organization (WHO) reported that there were approximately 324635 new cases (1.7% of all cancer cases) and 57043 deaths (0.6% of all cancer deaths) worldwide in 2020 [5]. Middle-aged people (>30 years old), especially in low- and middle-income countries, were more likely to be diagnosed with advanced cancer due to limited access to early diagnostic measures and suboptimal treatment [6, 7]. Due to limited treatment and census resources, population-based cancer screening in high-income areas may be less effective than in low- and middle-income areas [8]. Integrative cancer screening is a complicated method that requires consideration of biological and social variables, as well as ethical limitations. As previously stated, early detection of melanomas is associated with a better prognosis and survival [9]. Therefore, it is critical to identify the most accurate and reliable methods for detecting early symptoms.

Artificial Intelligence (AI) has recently altered the landscape of cancer research and medical oncology using traditional machine learning (ML) algorithms and cutting-edge deep learning (DL) architectures [10], especially in diagnosis (prostate cancer, breast cancer), patient classification (gastrointestinal cancer, melanoma, and skin cancer), and cancer prognosis and survival [11]. In addition, there is high variability in image processing by the operator, which affects the judgment of the clinicians and the instrument. Therefore, the processing of medical images is often prone to misjudgment but for many rare diseases; it is up to experienced clinicians to make the judgment call. Unlike experienced clinicians, it is a challenge for those in remote areas and with less experience to acquire accurate and correct information from medical images [12].

Deep learning is a field of artificial intelligence that uses artificial neural networks, a type of machine learning technique, to discover patterns and make predictions from massive datasets [13]. Machine learning offers considerable advantages for assimilating and evaluating large amounts of complex healthcare data [14]. Machine learning has ideal accuracy in early melanoma detection, and deep convolutional neural network offers the greatest classification accuracy among other techniques for machine learning.

In recent years, machine learning has been gradually introduced to distinguish between melanoma and benign nevus. To date, there are only a few systematic reviews of image classification applied to cancer to evaluate the diagnostic value of machine learning algorithms [15], especially in distinguishing melanoma from benign nevus. Therefore, this systematic review and meta-analysis were conducted to explore the accuracy of machine learning in the early diagnosis of melanoma.

## 2. Methods

### 2.1. Protocol Registration and Study Design

The study protocol was registered on the PROSPERO International register of systematic reviews (CRD42021252379). The study was conducted in accordance with the preferred reporting items for systematic reviews and meta-analyses (PRISMA) guidelines [13]. The current systematic review and meta-analysis did not require ethical approval or informed consent.

### 2.2. Search Strategy and Eligibility Criteria

Web of Science, Embase, PubMed, and the Cochrane library were searched for studies published until March 26, 2022. There were no restrictions on regions, languages, or publication types. Letters, scientific reports, scientific reports, scientific reports, animal experiments, conference abstracts, and narrative reviews were all excluded. The following search algorithm were used: (Melanoma OR meningioma OR malignant melanoma OR Nevus OR Mole OR PWS OR naevi) AND (Deep learning OR DL OR Convolutional neural network OR CNN OR Deep neural network OR Automated technique OR Artificial intelligent).

### 2.3. Inclusion and Exclusion Criteria

The inclusion criteria were as follows: (1) patients with fully documented melanoma or benign nevus were included in this systematic review; (2) ML and DL models are employed in medical imaging to diagnose melanoma and benign nevus; (3) the outcome measures, such as ROC, accuracy, specificity, and sensitivity, should be reported; (4) original studies including case-control study, cohort study, and nested case-control study were included; (5) there is no restriction on whether the machine learning of the original study has been externally validated. In addition, different machine learning models constructed from the same batch of data will also be included in our systematic review.

The exclusion criteria were as follows: (1) minor patients; (2) analysis of influencing factors and research on the incomplete classification model; (3) studies without the evaluation of the accuracy of machine learning; (4) systematic review, expert consensus, and conference summary; and (5) a systematic review on machine learning. In the original study, if the sample number is too small (for example, less than 50), there will be a certain bias in machine learning. Therefore, we need to exclude studies with too few samples.

### 2.4. Data Extraction

Li and Chu independently extracted the data on study characteristics and diagnostic performance using predetermined data extraction sheet. Any disagreements were resolved by Qi. Binary data relevant to diagnostic accuracy were immediately retrieved and input into contingency tables, which contained true positives, false positives, true negatives, and false negatives. These data were used to calculate pooled sensitivity, pooled specificity, and other outcome measures. If a study provided multiple contingency tables for the same or different DL/ML algorithms, we assumed that they were independent of each other.

### 2.5. Quality Assessment

The three investigators used the quality assessment of diagnostic accuracy studies 2 (QUADAS-2) tool to assess the risk of bias and applicability of the included studies [14]. The assessment tool consists of four items: patient selection, index tests, reference standards, and flow and time. Each item can be rated as high (red), unclear (yellow), or low (green) risk of bias. Likewise, the first three items assessed the applicability items and rated them as high, unclear, or low risk of bias.

### 2.6. Statistical Analysis

A random-effects model was used to assess the diagnostic performance of the algorithm, pool the effect values (95% confidence intervals (CI)), and evaluate the sensitivity and specificity of the algorithm. Meta-analysis was performed to investigate the best accuracy of the model in studies with multiple DL algorithms. Heterogeneity was measured, and subgroup analyses were conducted to explore the potential sources of heterogeneity.

Meta-analysis was only performed where there were more than or equal to three original studies. STATA (version 17.0) and RStudio (version 4.2.2) were used for data analyses. The threshold for statistical significance was set at *p* < 0.05.

## 3. Results

### 3.1. Study Selection

**Figure 1:**
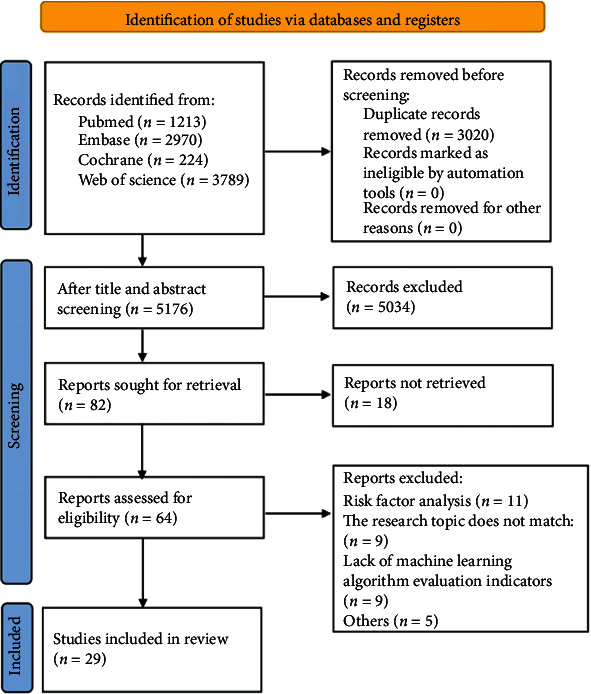
Flow chart for study selection process.

A total of 8196 records were identified initially. After removing 4176 papers for duplication and 3728 for failing to match the inclusion criteria, the full text of 142 studies was evaluated, with 112 articles being eliminated. Finally, data can only be extracted from 20 of the 29 studies that meet the criteria (see Figure 1).

### 3.2. Basic Characteristics of the Study Were Included

Among the included studies, 28 studies [16–42] were case-control studies and only one was a cohort study [43]. A total of 114007 participants were included, along with 71 machine learning models, with a convolutional neural network serving as the main machine learning method. There were 46 machine learning models reporting *c*-statistic, and 61 machine learning models could directly or indirectly calculate TP, FP, FN, and TN.

Six studies excluded low-quality images, while the remaining 23 studies did not report image quality. Two studies performed external validation using the out-of-sample dataset, while the others performed internal validation using the in-sample dataset. In six researches, the decisions of DL algorithms were compared to those of clinicians using the same dataset.

### 3.3. Risk of Bias Assessment

The quality of the included studies was assessed using QUADAS-2, and a detailed assessment for each item was provided. In terms of the risk of bias in patient selection, 12 studies were considered to have a high or unclear risk of bias due to unreported inclusion or exclusion criteria, as well as improper exclusion. For the risk of bias in the index test, four studies were deemed to have a high or unclear risk of bias in the index test due to the lack of a set threshold.

For the risk of bias in the reference standard, two studies were deemed to have a high or unclear risk of bias due to inconsistencies in the reference standard. It was not stated whether the threshold was set in advance or whether blinding was used. Furthermore, six studies were assessed to have a high or unclear risk of bias in flow and timing because the studies did not state whether there was an appropriate time gap or whether it was based on the same standard. There were 11 studies considered to have high or unclear applicability in patient selection. One study had unclear applicability in the reference standard. There was no applicability concern in the index test.

### 3.4. Pooled Performance of DL/ML Algorithms

Among the 30 studies in this sample, 46 machine learning models reported *c*-statistic, and 61 machine learning models could directly or indirectly calculate TP, FP, FN, and TN. Most studies used more than one DL algorithm to report diagnostic performance. The combined *C*-index was 0.87 (95% CI: 0.84-0.90) for 46 machine learning models. (The results are shown in Table 1.)

**Figure 2:**
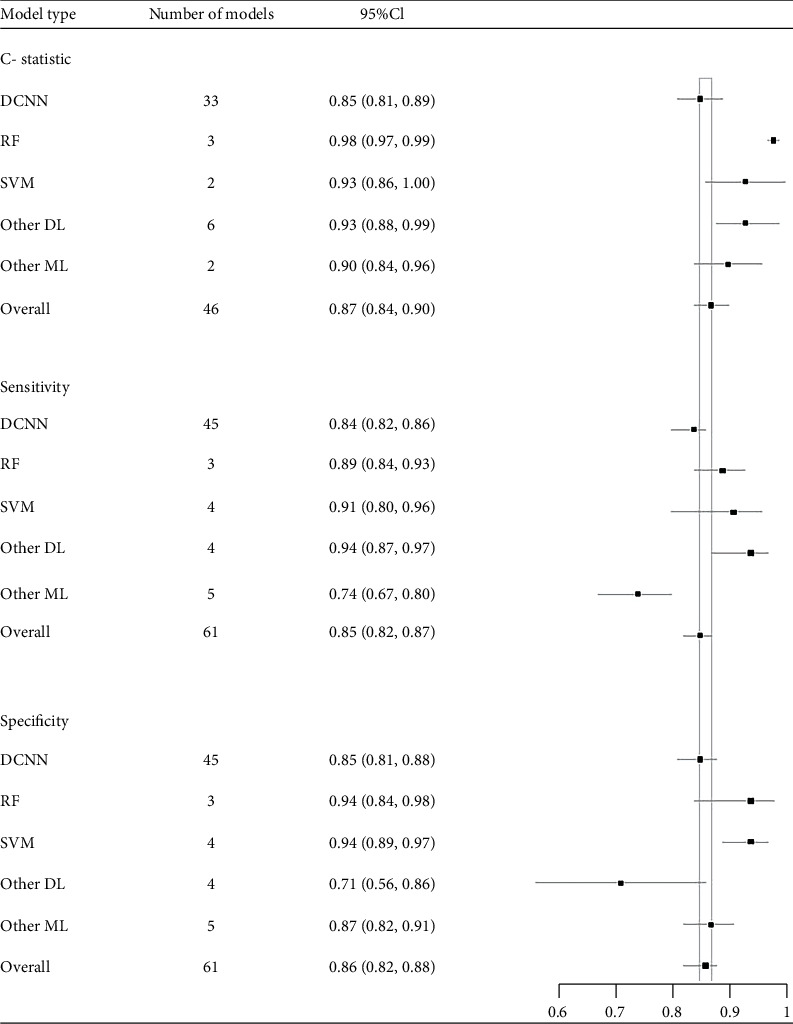
Forest maps of C-statistic, sensitivity, and specificity of different machine learning types.

The combined sensitivity and specificity of 61 outcome indicators were 85% (95% CI: 82-87) and 86% (95% CI: 82-88), respectively (Figure 2).

### 3.5. Subgroup Analysis

There were 46 machine learning models reporting the c-statistic of the model. The results of *c*-statistic of each kind of machine learning were as follows: DCNN—0.85 (95% CI: 0.81-0.89), RF—0.98 (95% CI: 0.97-0.99), SVM—0.93 (95% CI: 0.86-1.00), other DL—0.93 (95% CI: 0.88-0.99), and other ML—0.92 (95% CI: 0.87-0.96).

The sensitivity and specificity of 61 machine learning models in diagnosing melanoma were examined. The results of the combined sensitivity of each machine learning type were as follows: DCNN—0.84 (95% CI: 0.82-0.86), RF—0.89 (95% CI: 0.94-0.93), SVM—0.91 (95% CI: 0.91-0.96), other DL—0.94 (95% CI: 0.87-0.97), and other ML—0.74 (95% CI: 0.67-0.79). The results of total specificity were as follows: DCNN—0.85 (95% CI: 0.81-0.88), RF—0.94 (95% CI: 0.84-0.98), SVM—0.94 (95% CI: 0.89-0.97), other DL—0.71 (95% CI: 0.71), and other ML—0.87 (95% CI: 0.83-0.91) (see Table 1 and Figure 2).

### 3.6. Heterogeneity Analysis

As demonstrated by all of the included studies, DL/ML algorithms outperformed histopathological analysis in distinguishing benign moles and melanomas in medical images. However, the heterogeneity was significant. First, the unique machine learning system evaluation resulted in diversification. Secondly, in order to effectively classify melanoma and benign nevus, different effective outcome measures were used, which was the main source of the heterogeneity. Furthermore, there were differences between the researchers during adjustment even in the same model, which could also generate heterogeneity.

## 4. Discussion

This study systematically evaluated the effects of using machine learning in distinguishing melanoma and benign nevus in demography. Methodologically, machine learning studies can be divided into ML and DL. Since 2019, ML has made a significant transition to DL and accumulating research focused on deep learning in identifying melanoma and benign nevus. Therefore, a systematic review of published studies on machine learning is necessary to provide guidance for future research. The sample size in the ML research was found to be larger than in the DL studies. However, there was no statistically significant difference in heterogeneity between the ML and DL groups. Based on the improved PROBAST, we found that some studies had a high or undetermined risk of bias in patient selection, reference criteria, and timing. These results revealed current technology and the necessity for quality improvement.

Through the systematic search for relevant articles, we found three systematic reviews and meta-analyses which investigated the application of DL algorithms in medical imaging [41, 42, 44]. However, at present, most research investigated the recognition of melanoma based on machine learning, and there was no systematic review on distinguishing melanoma from benign nevus based on machine learning. According to the research of Haggenmüller et al. [45], the performance of classifiers based on CNN was better or at least comparable to that of clinicians. However, almost all studies were performed in highly controlled environments and relied entirely on single images of suspected lesions.

Cui et al. [46] found that DL algorithms demonstrated better performance in diagnosing. However, the authors also discovered high heterogeneity, which they attributed to combining distinct methods and perhaps through unspecified terms. Previous research showed that the diagnostic accuracy of DL should be considered with caution, and there was a need to develop (and apply) AI guidelines. These conclusions were consistent with our study.

Although 30 papers matched the inclusion criteria for the systematic review, only 15 of them had data that could be utilized for contingency tables. Some algorithm research published in computer science journals only reported precision, dice coefficient, F1 score, recall, and competition performance metric, but not AUC, accuracy, sensitivity, and specificity. Bridging the gap between computer sciences research would seem prudent if we are to conduct interdepartmental research and transition to a more digitized healthcare system. Moreover, we found the term “validation” is used causally in studies on the DL model. Some authors characterized it as a dataset for model tuning during the development process, while others defined it as a dataset for assessing the diagnostic performance of the final algorithm. This confused readers and made determining the function of datasets difficult. We referred to relevant literature and proposed the dataset used in the development and validation of the discriminating algorithm. Due to the small number of validation sets, we only included training sets and test sets in the study.

Four of the 30 included studies showed that machine learning demonstrated better performance compared to clinicians in distinguishing melanoma from a benign nevus, but DL/ML-based models were susceptible to confounding factors [22, 23, 30, 36].

Most studies focused on diagnostic performance in reporting algorithms rather than clinical practice, which resulted in poor reporting and less authenticity in clinical practice. Furthermore, this resulted in inadequate data availability, limiting us from evaluating the application of these algorithms in clinical practice [47]. Therefore, a double check by professionals is of importance to avoid false diagnoses. Thus, until artificial intelligence is completely developed, it is more like an auxiliary tool.

We analyzed the research with diagnostic classification tasks as the main objective. However, this is only one of the aspects of improving personalized patient care. In addition to using AI-based assistance systems to distinguish between the two diseases, further improving precision medicine and treatment options are the focus of future development [48]. We should consider not only a comparison of computer-assisted diagnosis studies but also research advancement on prognostic endpoints.

Finally, because research with positive or statistically significant outcomes was more likely to be published than studies with negative results, bias might have developed. In addition, different models result in different parameters, outcomes in identifying images, and time spans, all of which were sources of bias. The risk of publication bias could not be excluded.

## 5. Conclusions

Our paper summarized the methodological characteristics, calibration, and performance of current algorithms in distinguishing melanoma and benign nevus. There are several algorithm models available to clinicians and researchers, but using relative models in specific environments and populations might be challenging owing to different tuning decisions. Future research should focus on calibrating models or updating existing models.

According to our research, only a few studies have carried out test verification and internal and external verification on the model. Test and verification sets are required in further studies to fully estimate the performance and calibration measures of model adjustment.

In addition, validation studies on setting and including criteria in different populations are essential for ensuring classification model generality. In clinical practice, classification models with appropriate calibration and external validity should be used to examine the effect on specific outcomes.

## Figures and Tables

**Table 1:** Summary of specificity and *C*-index in subgroup analysis.

E	Model	c-statistic		Sensitivity and specificity		
		Number	95% CI	Number	SEN (95% CI)	SPE (95% CI)
1	DCNN	33	0.85 (0.81, 0.89)	45	0.84 (0.82, 0.86)	0.85 (0.81, 0.88)
2	RF	3	0.98 (0.97, 0.99)	3	0.89 (0.84, 0.93)	0.94 (0.84, 0.98)
3	SVM	2	0.93 (0.86, 1.00)	4	0.91 (0.80, 0.96)	0.94 (0.89, 0.97)
4	Other DL	6	0.93 (0.88, 0.99)	4	0.94 (0.87, 0.97)	0.71 (0.56, 0.86)
5	Other ML	2	0.90 (0.84, 0.96)	4	0.74 (0.67, 0.80)	0.87 (0.82, 0.91)
Overall		46	0.87 (0.84, 0.90)	61	0.85 (0.82, 0.87)	0.86 (0.82, 0.88)

In the original study, other DL was described as a combination of various artificial neural networks; other ML contained KNN and LR.

## Data Availability

The data that support the findings of this study are available from the corresponding author upon reasonable request.
